# Effective treatment of recalcitrant palmoplantar psoriasis in a patient with skin of color using bimekizumab

**DOI:** 10.1016/j.jdcr.2025.06.020

**Published:** 2025-06-24

**Authors:** Emily G. Tocco, Margaret G. Mercante, Diego Ruiz Dasilva

**Affiliations:** a University of Virginia School of Medicine, Charlottesville, Virginia; b Forefront Dermatology, Eastern Virginia Medical School, Virginia Beach, Virginia

**Keywords:** bimekizumab, interleukin-17 inhibitors, palmoplantar psoriasis, refractory psoriasis, skin of color

## Introduction

Palmoplantar psoriasis is a localized form of psoriasis primarily affecting the palms and soles, often causing significant itching, pain, and functional impairment. This disease is characterized by well-defined erythematous plaques with scaling and hyperkeratosis, occasionally accompanied by pustules, and accounts for approximately 3% to 4% of all psoriasis cases.^1^ Histopathology of palmoplantar psoriasis reveals hyperkeratosis, parakeratosis, regular acanthosis, and a mixed inflammatory infiltrate with lymphocyte predominance.^2^ Pathways of inflammation differ between pustular and nonpustular subtypes, with the gamma interferon pathway being more active in the nonpustular cases.^3^ Management usually begins with topical therapies, such as corticosteroids or vitamin D analogs, with systemic treatments such as methotrexate and biologic agents targeting tumor necrosis factor alpha, interleukin (IL)-17, and IL-23 used for more severe cases.^1^

In patients of color, palmoplantar psoriasis may present with more pronounced hyperpigmentation and less erythema, making it potentially difficult to differentiate from similarly presenting conditions, including lichen planus and tinea corporis. In these cases, a skin biopsy may be helpful to elucidate the correct diagnoses.

## Case report

**Fig 1:**
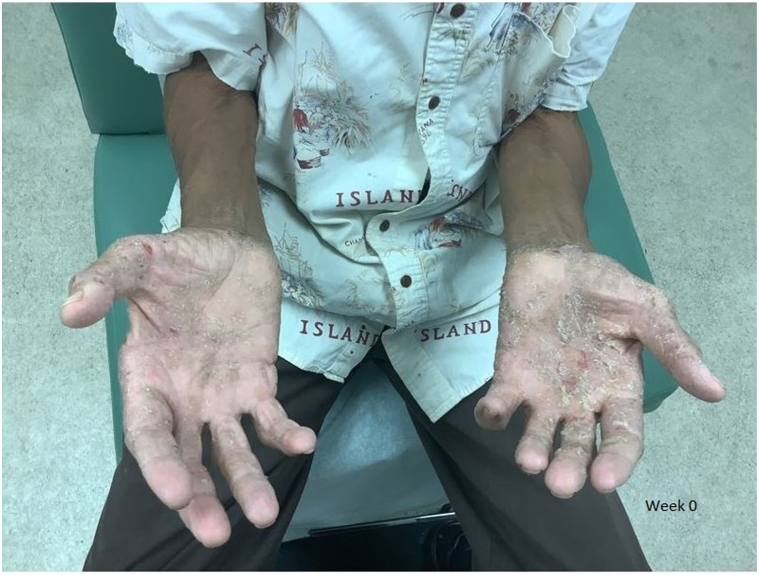
Psoriatic plaques on the patient’s palms prior to treatment, showing thick scaling and erythema.

A 78-year-old African American male with a history of palmoplantar psoriasis, hypertension, hyperlipidemia, type 2 diabetes mellitus, and coronary artery disease presented with an 8-month history of worsening itchy, painful, and scaly plaques and fissures involving the palmoplantar surfaces as well as joint discomfort (Fig 1).

The patient failed numerous treatments, including topical therapies such as triamcinolone 0.1% cream, clobetasol 0.05% ointment, tapinarof cream, and roflumilast cream, as well as biologic agents including secukinumab and adalimumab. Despite these efforts, his condition showed no improvement. The patient was also placed on 4 courses of prednisone in 8 months, which temporarily alleviated his skin and joint symptoms but led to rebound flares once tapered.

**Fig 2:**
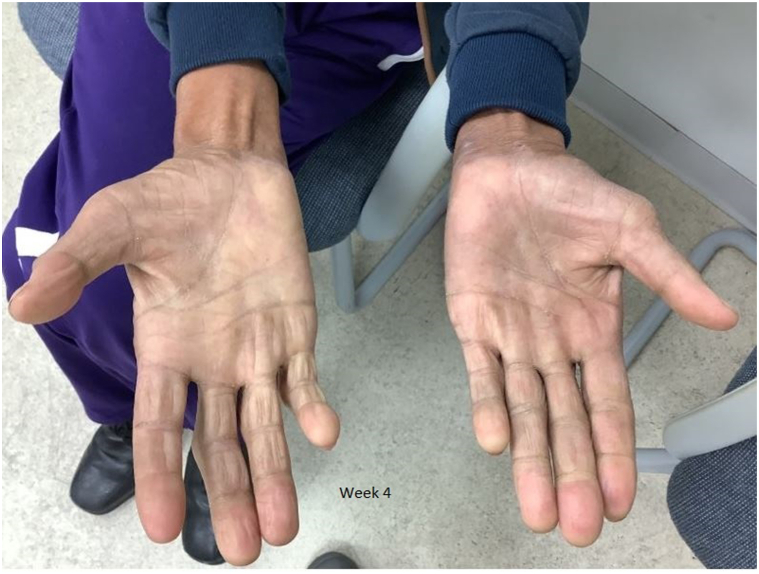
Marked improvement in the same patient’s palms after 4 weeks of treatment with bimekizumab, demonstrating near-complete resolution of plaques.

Baseline laboratory investigations, including complete blood count, comprehensive metabolic panel, hepatitis panel, HIV testing, and QuantiFERON-TB Gold, were all normal. Biopsy of right palmar skin revealed spongiotic and psoriasiform dermatitis favoring psoriasis. With no contraindications identified, the patient was started on 320 mg of bimekizumab, an IL-17A and IL-17F inhibitor, administered every 4 weeks. At his 1-month follow-up, he reported dramatic improvement, with significant resolution of scaling and cracking skin and near-complete clearance of plaques (Fig 2). Additionally, his itching and joint discomfort had markedly improved, and he experienced no adverse effects.

## Discussion

Palmoplantar psoriasis is a chronic inflammatory condition affecting the hands and feet, often resulting in severe discomfort and functional challenges. This case highlights the challenges of managing intractable palmoplantar psoriasis and the limitations of conventional treatments. The patient's rapid and sustained response to bimekizumab underscores the therapeutic potential of selectively targeting interleukin pathways, particularly IL-17A and IL-17F, in refractory cases. Notably, the patient had shown minimal response to secukinumab, which targets IL-17A alone, but experienced marked clinical improvement with bimekizumab, which inhibits both IL-17A and IL-17F. This differential response highlights the potential contribution of IL-17F to disease activity in certain patients and supports the rationale for broader cytokine blockade in refractory cases. Furthermore, this case demonstrates the risks associated with prolonged corticosteroid use, which may offer only temporary relief while exacerbating rebound symptoms.

In patients with skin of color, the subtle presentation of erythema, which can appear violaceous, gray, or brown, can make psoriasis diagnosis more challenging.^4^ Additionally, treatment is often complicated by an increased risk of postinflammatory hyperpigmentation associated with corticosteroid use.^5^ Bimekizumab’s strong efficacy and favorable safety profile make it a particularly promising option for skin of color patients, who may present with unique immune responses and risk factors.^6^^,^^7^

## Conflicts of interest

None disclosed.

## References

[1] Engin B., Aşkın Ö., Tüzün Y. (2017). Palmoplantar psoriasis. Clin Dermatol.

[2] Das G., Mathur M., Shrestha A., Jaiswal S., Maharjan S. (2024). Palmoplantar psoriasis: a clinicopathological correlation in a tertiary care hospital. Skin Res Technol.

[3] Wang C.Q., Haxhinasto S., Garcet S. (2023). Comparison of the inflammatory circuits in psoriasis vulgaris, non‒pustular palmoplantar psoriasis, and palmoplantar pustular psoriasis. J Invest Dermatol.

[4] Khanna R., Khanna R., Desai S.R. (2023). Diagnosing psoriasis in skin of color patients. Dermatol Clin.

[5] Hussain M., Khan R., Khalil A. (2021). Post-inflammatory hyperpigmentation in dermatologic therapy: a review. Int J Dermatol.

[6] Saurat J.H., Smith C.H., Puig L. (2020). Efficacy and safety of bimekizumab in patients with moderate-to-severe psoriasis: results from a phase 3 trial. Lancet.

[7] Armstrong A.W., Read C., Papp K.A. (2021). Bimekizumab in moderate-to-severe psoriasis: a review of recent studies. J Dermatol Ther.

